# Adrenergic Agonists Bind to Adrenergic-Receptor-Like Regions of the Mu Opioid Receptor, Enhancing Morphine and Methionine-Enkephalin Binding: A New Approach to “Biased Opioids”?

**DOI:** 10.3390/ijms19010272

**Published:** 2018-01-17

**Authors:** Robert Root-Bernstein, Miah Turke, Udaya K. Tiruttani Subhramanyam, Beth Churchill, Joerg Labahn

**Affiliations:** 1 Department of Physiology, Michigan State University, 567 Wilson Road, Room 2201 Biomedical and Physical Sciences Building, East Lansing, MI 48824, USA; turkemia@msu.edu (M.T.); church49@msu.edu (B.C.); 2 Centre for Structural Systems Biology (CSSB), Notkestraße 85, 22607 Hamburg, Germany; udaya.tiruttani@desy.de (U.K.T.S.); j.labahn@fz-juelich.de (J.L.); 3 Forschungszentrum Juelich GmbH, ICS-6, 52425 Juelich, Germany

**Keywords:** biased opioids, morphine, methionine-enkephalin, epinephrine, norepinephrine, enhancement, synergy, allosteric, mu opioid receptor, receptor dimers, dimerization

## Abstract

Extensive evidence demonstrates functional interactions between the adrenergic and opioid systems in a diversity of tissues and organs. While some effects are due to receptor and second messenger cross-talk, recent research has revealed an extracellular, allosteric opioid binding site on adrenergic receptors that enhances adrenergic activity and its duration. The present research addresses whether opioid receptors may have an equivalent extracellular, allosteric adrenergic binding site that has similar enhancing effects on opioid binding. Comparison of adrenergic and opioid receptor sequences revealed that these receptors share very significant regions of similarity, particularly in some of the extracellular and transmembrane regions associated with adrenergic binding in the adrenergic receptors. Five of these shared regions from the mu opioid receptor (muOPR) were synthesized as peptides and tested for binding to adrenergic, opioid and control compounds using ultraviolet spectroscopy. Adrenergic compounds bound to several of these muOPR peptides with low micromolar affinity while acetylcholine, histamine and various adrenergic antagonists did not. Similar studies were then conducted with purified, intact muOPR with similar results. Combinations of epinephrine with methionine enkephalin or morphine increased the binding of both by about half a log unit. These results suggest that muOPR may be allosterically enhanced by adrenergic agonists.

## 1. Introduction

Opioid receptors mediate nociception and analgesia in a manner that is integrally linked with catecholamine function. Agonists of the α-2A adrenergic receptor (α2A-ADR) and the mu opioid receptor (muOPR) jointly modulate analgesia [1] in the nervous system and autonomic functions [2], especially those involving the cardiovascular and gastrointestinal systems [3,4]. Anatomically, α2A-adrenergic receptors are co-localized within various neurons or are expressed on adjoining neurons that share synapses [5,6]. Opioid peptides and catecholamines are co-stored and co-released in neurons and the adrenals [7,8,9,10,11], again suggesting integration of the two systems at the most basic levels. There are even direct functional interactions between ADR and OPR resulting from co-localization and dimerization of the receptors in the cell membrane [12,13,14,15,16,17,18,19,20]. Co-functionality is such that ADR control the locomotor and reward effects of opioids [21] and knocking out α1b-adrenergic receptors and 5-HT2A receptors simultaneously not only eliminated the response of mice to amphetamine and cocaine, but also to morphine [22].

Integration of opioid and adrenergic function extends even to their individual receptors. Opioids bind to receptors other than their own [23], a difficulty that continues to plague opiate drug development [24,25], and ADR are among these. Opioids and, notably, opioid antagonists both enhanced activation of ADR at any sub-maximal dose of the adrenergic drug; the duration of activity of the adrenergic drug is also greatly increased [26,27,28,29,30,31,32,33,34,35,36,37,38,39,40,41]. Munro et al. [24], Root-Bernstein and Dillon [42,43,44] demonstrated that morphine, met-enkephalin and naloxone each bind to adrenergic receptors at sites located in their extracellular loops. Thus, integration of opioid enhancement of adrenergic activity is literally integrated into the structure of ADR.

Clinical and experimental studies suggest that a parallel enhancement of opioid receptors (OPR) by adrenergic agonists (but, notably, not antagonists) also exists. “Opioid and α2-adrenoceptor (AR) agonists are analgesic when administered in the spinal cord and show a clinically beneficial synergistic interaction when co-administered”, according to Chabot-Doré et al. [20]. Many other studies have confirmed such an effect for mu and delta OPR as well as for a wide range of adrenergic agonists ranging from amphetamines to clonidine (e.g., [45,46,47,48,49,50,51,52,53,54,55,56,57,58]). The enhancement of opioid function by adrenergic agonists, particularly amphetamines, has been observed in other systems as well, such as opioid-mediated reward and related behaviors [59,60,61,62,63,64,65]; glucose uptake in the brain [66]; and on guinea pig ileum contractions induced by morphine, which are also enhanced by serotonin [67,68,69].

Given the multiple levels of integration of adrenergic and opioid functions, and particularly the existence of opioid binding sites on adrenergic receptors, we wondered whether a corresponding allosteric adrenergic binding site for adrenergic (and perhaps serotoninergic) compounds might exist on opioid receptors that might enhance opioid activity. This paper reports an initial investigation of this possibility and explores the mechanistic implications of an allosteric mechanism for understanding opioid-adrenergic synergy.

## 2. Results

**Figure 1:**
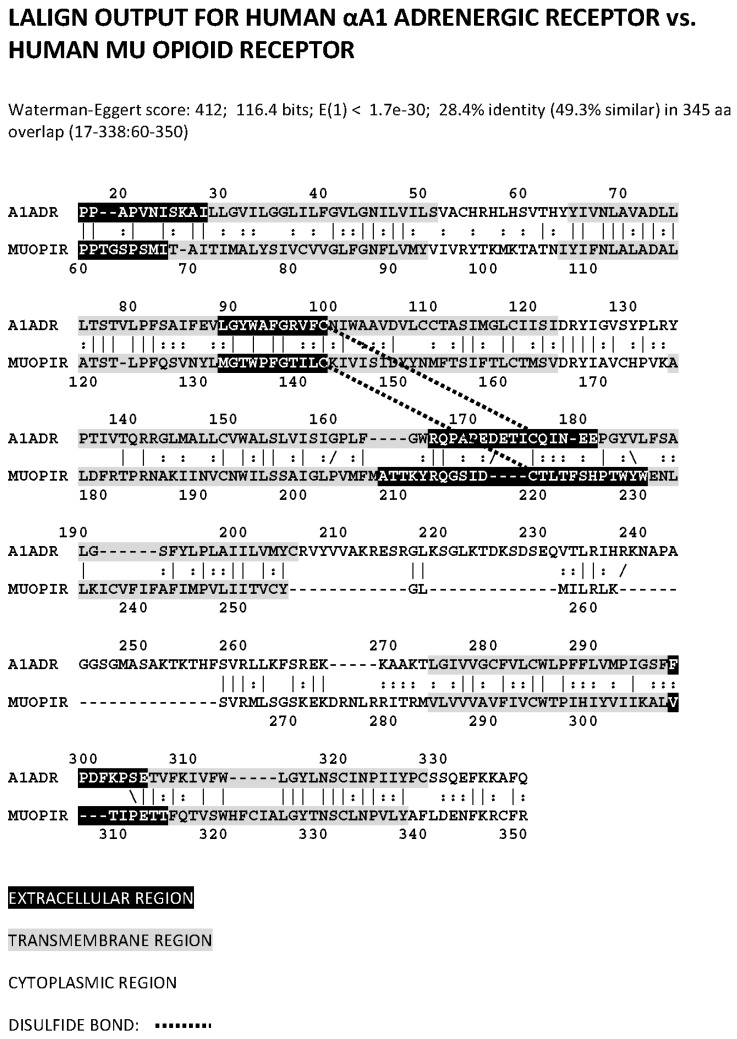
LALIGN (Available online: www.expasy.org) similarity comparison of the human α A1 adrenergic receptor (A1ADR) with the human mu opioid receptor (muOPR) displaying sequence similarities that exist primarily in the first extracellular loop (A1ADR 90–100) and second extracellular loop (A1ADR 165–582) regions (displayed in white lettering on black background), and in the flanking transmembrane regions. Bars represent amino acid identities as do back- or forward slashes; double dots represent similar amino acids. The dashed lines indicate disulfide bonds between cysteine residues. Notably, the third extracellular loop exhibits no significant similarity (A1ADR 297–306), nor do most of the cytoplasmic regions of the two receptors.

We began our investigation of whether opioid receptors might have adrenergic binding capacity by determining whether the αA1 adrenergic (ADR) and mu opioid receptors (muOPR) have sequence similarity. The two receptor types are highly homologous, being almost 50% identical across their entire sequences, with some regions approaching 75% similarity (Figure 1). Notably, the regions of highest similarity, which comprise the first and second extracellular loops and their corresponding transmembrane sequences, are associated with ligand binding in adrenergic receptors. These sequences appear to be very highly conserved between opioid and adrenergic receptors across species, but these data and their evolutionary implications will be reported elsewhere.

**Figure 2:**
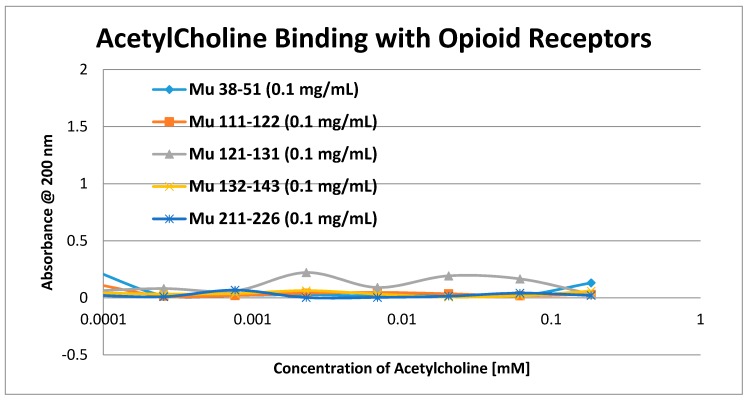
UV spectroscopic study of acetylcholine binding to mu opioid receptor (muOPR) peptides derived from the extracellular loop and adjacent transmembrane regions (see Figure 1).

**Figure 3:**
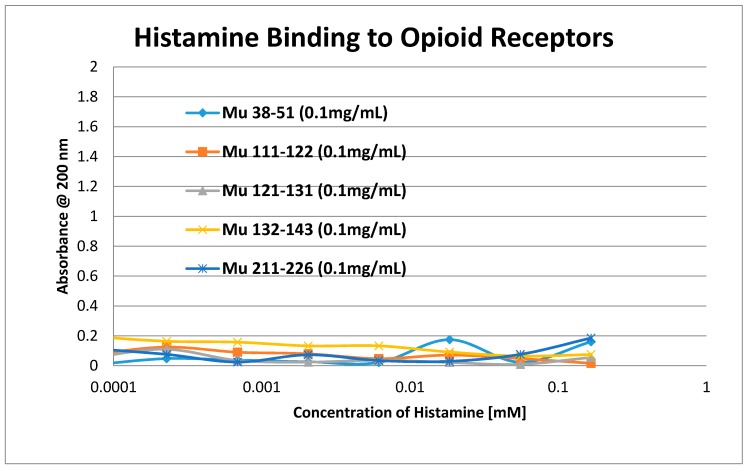
UV spectroscopic study of histamine binding to mu opioid receptor (muOPR) peptides derived from the extracellular loop and adjacent transmembrane regions (see Figure 1).

**Figure 4:**
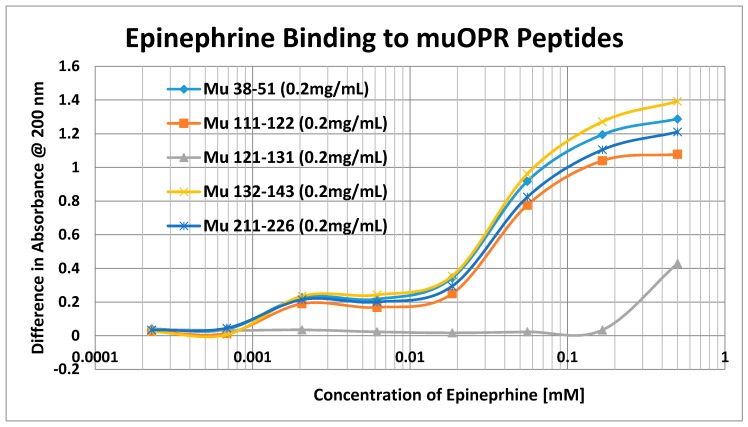
UV spectroscopic study of epinephrine binding to mu opioid receptor (muOPR) peptides derived from the extracellular loop and adjacent transmembrane regions (see Figure 1).

**Figure 5:**
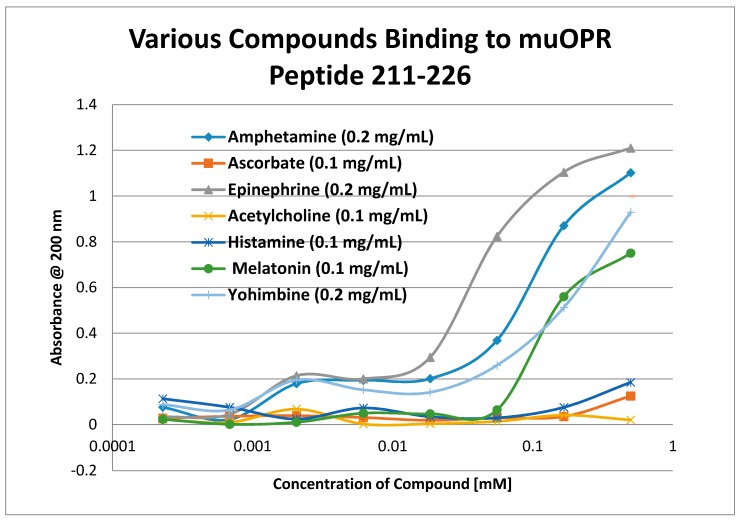
UV spectroscopic study of the binding of epinephrine, amphetamine, ascorbic acid (vitamin C), acetylcholine, yohimbine, and melatonin to mu opioid receptor (muOPR) peptide 211–226 derived from the second extracellular loop of the receptor (see Figure 1).

**Figure 6:**
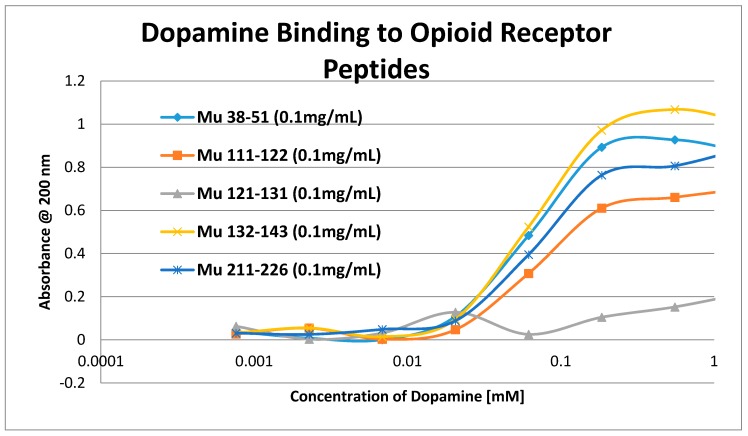
UV spectroscopic study of dopamine binding to mu opioid receptor (muOPR) peptides derived from the extracellular loop and adjacent transmembrane regions (see Figure 1).

**Figure 7:**
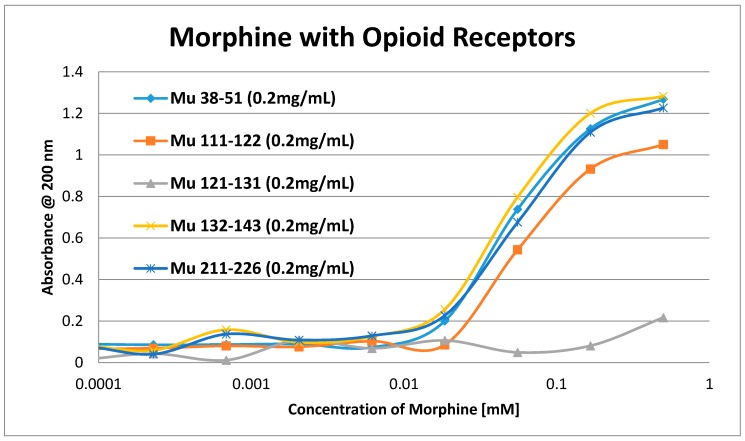
UV spectroscopic study of morphine binding to mu opioid receptor (muOPR) peptides derived from the extracellular loop and adjacent transmembrane regions (see Figure 1).

**Figure 8:**
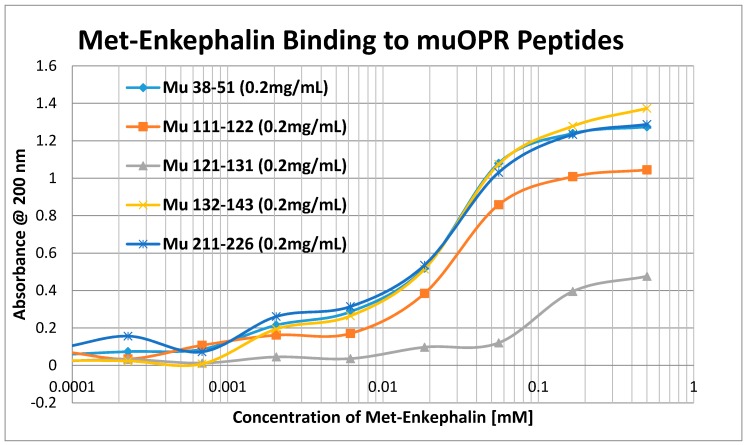
UV spectroscopic study of methionine enkephalin binding to mu opioid receptor (muOPR) peptides derived from the extracellular loop and adjacent transmembrane regions (see Figure 1).

Having identified similar regions shared by opioid and adrenergic receptors, we had five of the corresponding muOPR sequences synthesized as peptides as well as peptides from corresponding extracellular loops from other G-protein coupled receptors (Table 1). These peptides were then tested for binding to morphine, met-enkephalin, naloxone and various neurotransmitters and their agonists and antagonists to determine whether any of them might represent ligand binding regions appropriate for adrenergic enhancement of opioid receptor function. Binding was determined by UV spectroscopy, a method that we and other laboratories have previously validated by comparison with nuclear magnetic resonance imaging, circular dichroism studies and capillary electrophoresis [70,71,72,73,74,75,76,77,78,79]. Acetylcholine and histamine (Figure 2 and Figure 3) showed no measurable binding to the muOPR peptides. Epinephrine bound to four of the five muOPR peptides (Figure 4), displaying biphasic high-affinity (Kd = 1 to 2 μM) and low affinity (Kd = 30 to 40 μM) components that were present in a number of other adrenergic antagonists such as norepinephrine (Table 2) and amphetamine (Figure 5), but not dopamine (Figure 6), which only displayed the low affinity binding. Several of the muOPR peptides also bound morphine (Figure 7) with a low-affinity (Kd = 30 to 50 μM) component, and met-enkephalin bound to the same peptides (Figure 8) but with both a high affinity (Kd = 15 nM to 1 μM) and lower affinity (Kd = 55 to 90 μM) components. Notably, met-enkephalin also had some affinity for muOPR peptide 121–131 (a transmembrane region), which no other compound other than etheylenediaminetetracetic acid (EDTA) and serotonin (5-hydroxytryptamine) displayed (Table 2), indicating that it binds to the muOPR differently than does morphine. Binding constants of opioids to the opiate receptor were generally hundreds to thousands of times lower than to other receptors, while serotonin and melatonin generally displayed significantly lower affinity to the peptides than did adrenergic compounds (Table 2). One surprising result was that ascorbic acid (vitamin C), which binds with significant affinity to aminergic receptors [42,43] did not bind to muOPR peptides (Figure 5 and Table 2). The results of these and many control compounds are summarized in Table 2, which demonstrates that binding to muOPR peptides is generally limited to adrenergic agonists, serotonin agonists, and opioids, and that opioids have higher affinity (lower Kd’s) for OPR peptides compared with the extracellular loop peptides of other receptors. Adrenergic antagonists such as propranalol, yohimbine and phentolamine also had significantly lower affinity for muOPR extracellular peptides than did adrenergic agonists (Figure 5 and Table 2).

**Figure 9:**
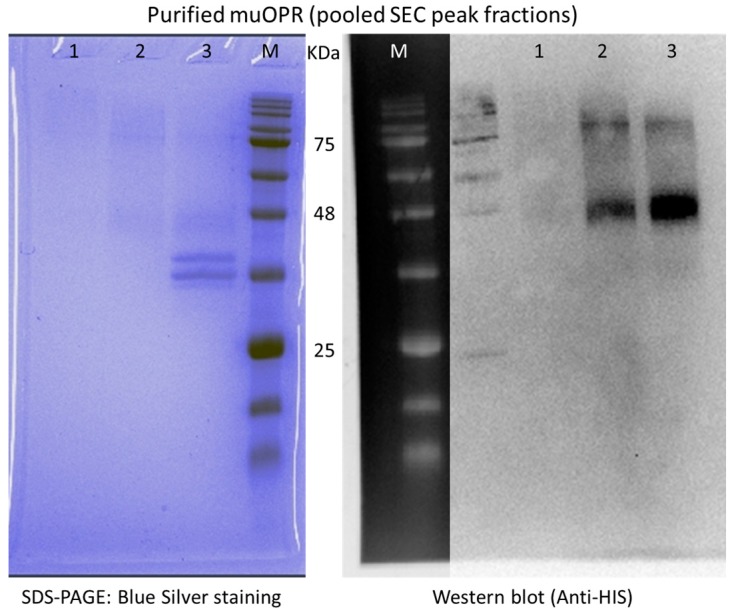
SDS-PAGE (blue silver staining) and Western blot (anti-HIS tag staining) of the eluted fractions of mu opioid-receptor obtained from HiLoad 16/600 superdex 200 pg column demonstrating the presence of the receptor (see Materials and Methods). Lane M is BlueElf prestained protein marker. The muOPR sample used for binding measurements is from Lane 2.

**Figure 10:**
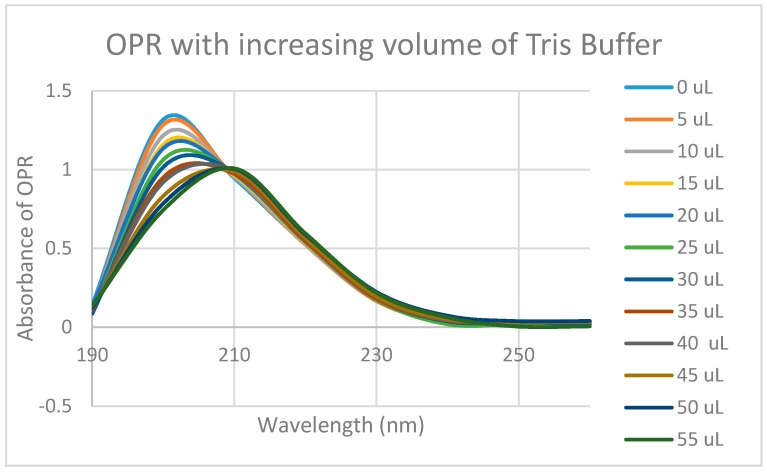
UV spectra of mu opioid receptor (OPR) with the same serial additions of Tris buffer in which the experiments illustrated in Figure 12, Figure 13, Figure 14, Figure 16 and Figure 17 were performed. Note that the absorbance at 210 nm does not change as a result of these additions, so 210 nm was used to analyze binding affinity due to addition of epinephrine, morphine and methionine-enkephalin in Figure 15 and Figure 18. Binding affinity was also calculated at 200 nm by accounting for buffer effects, but as a result, the calculated binding constants are somewhat less reliable (see Table 3).

**Figure 11:**
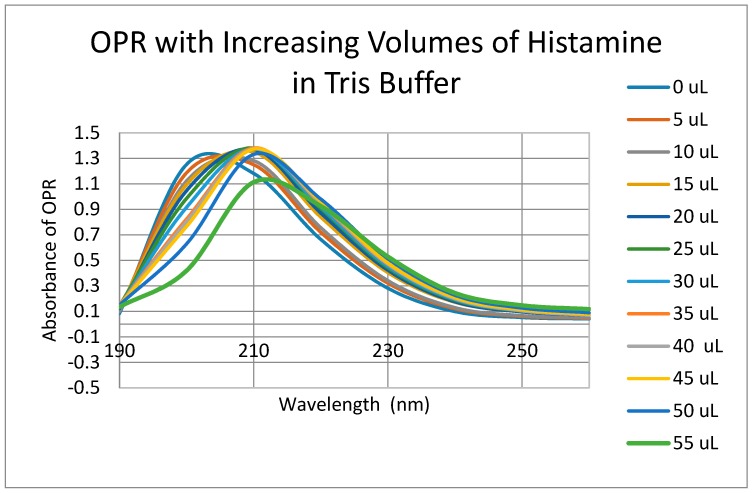
UV spectra of mu opioid receptor (OPR) with serial additions of histamine in Tris buffer as in Figure 10. No significant binding was observed for histamine or for acetylcholine (Table 3) or ascorbic acid (Table 3).

**Figure 12:**
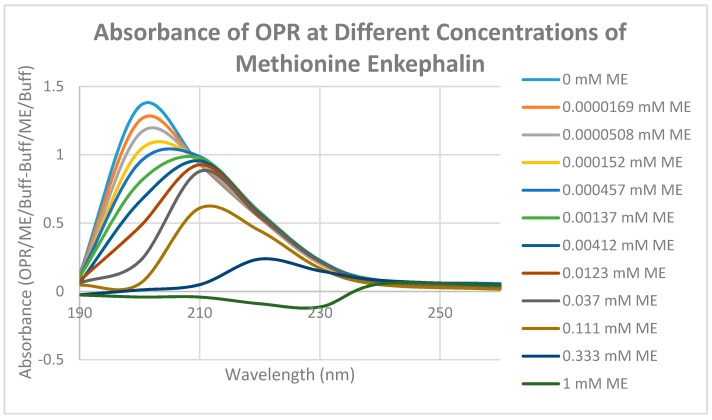
UV spectra of mu opioid receptor (OPR) with serial additions of methionine enkephalin (Met-Enk). Note the very significant differences between these spectra and those in Figure 10 and Figure 11.

**Figure 13:**
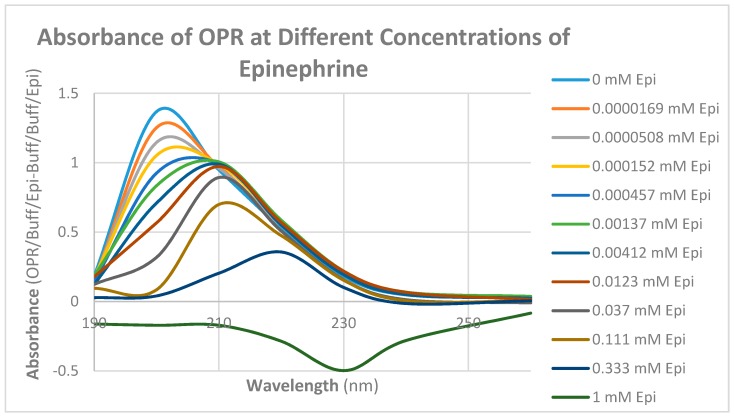
UV spectra of mu opioid receptor (OPR) with serial additions of epinephrine HCl (Epi). Note the very significant differences between these spectra and those in Figure 10 and Figure 11.

**Figure 14:**
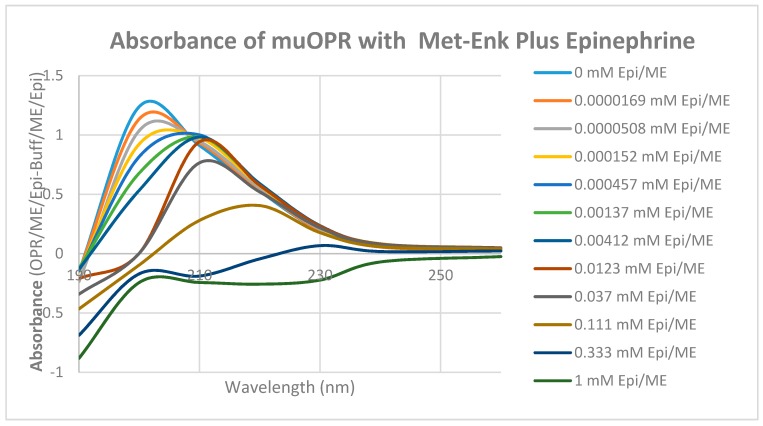
UV spectra of mu opioid receptor (OPR) with serial additions of both met-enkephalin (Met-Enk) and epinephrine (Epi) in tandem. Note the obvious differences from Figure 12 and Figure 13.

**Figure 15:**
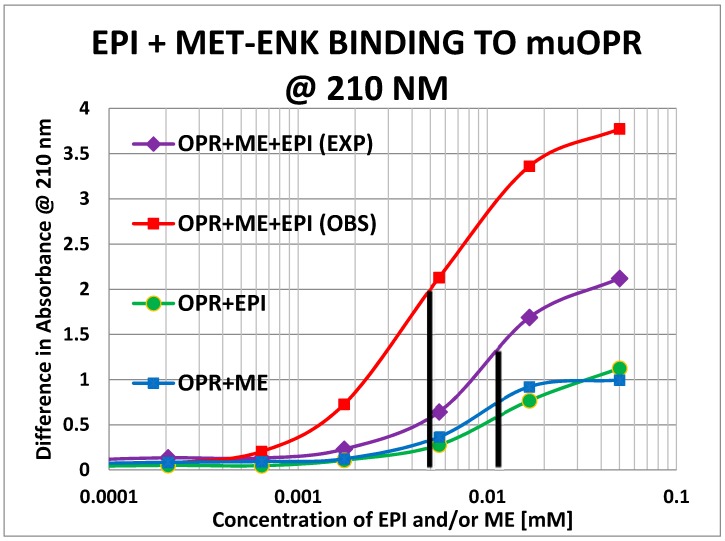
Mu opioid receptor (OPR) binding curves with methionine-enkephalin (ME), epinephrine (EPI) and their combination (ME + EPI) at 210 nm. The choice of 210 nm is explained in Figure 10. The experimentally observed binding curve (OPR + ME + EPI (OBS)) is compared with the theoretically predicted binding calculated from individual binding of ME to OPR and EPI to OPR (OPR + ME + EPI (EXP)). There is a half-log unit shift to the left (black vertical lines) in the observed binding as compared with the predicted binding (12 μM versus 5 μM).

**Figure 16:**
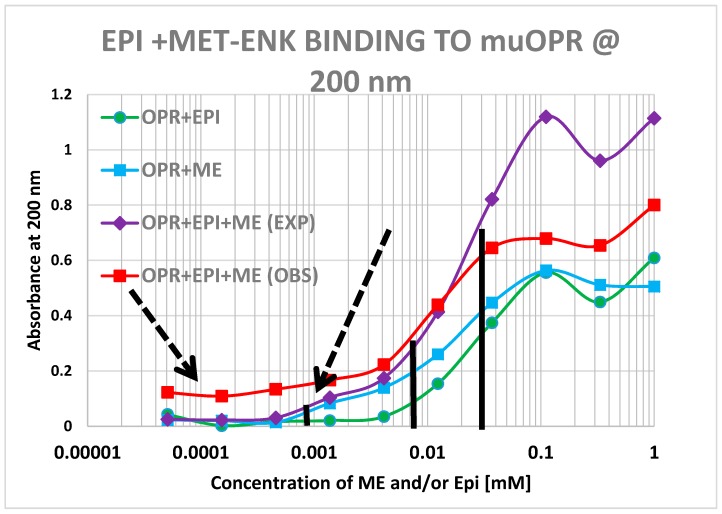
Mu opioid receptor (OPR) binding curves with methionine-enkephalin (ME), epinephrine (EPI) and their combination (ME + EPI) at 200 nm. There is, as at 210 nm (Figure 15) still a shift to the left (black vertical lines) in the observed binding as compared with the predicted binding (30 μM versus 10 μM). At 200 nm, however, the presence of high affinity binding of ME to the muOPR is also evident, with a binding constant of about 900 nM and there appears to be a dramatic increase in high affinity binding when both EPI and ME are present (dashed arrows).

**Figure 17:**
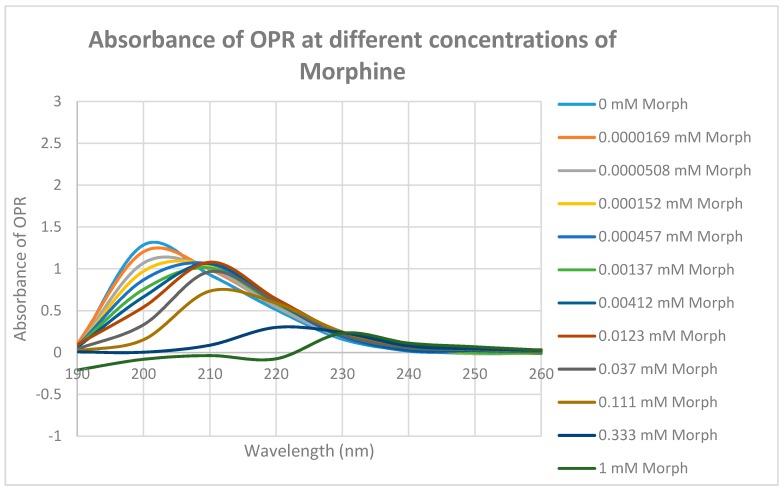
UV spectra of mu opioid receptor (OPR) with serial additions of morphine. Note the very significant differences between these spectra and those in Figure 10 and Figure 11.

Since some muOPR peptides bound adrenergic compounds, intact, purified mu opioid receptor was tested for its ability to bind opioid and adrenergic compounds. Purified human muOPR was expressed in *E. coli* and purified (Figure 9) as described in Materials and Methods [80,81]. Binding of compounds was again characterized using UV spectroscopy (Figure 10, Figure 11, Figure 12, Figure 13, Figure 14, Figure 15, Figure 16 and Figure 17). Since aliquots of each compound were added serially, and very small changes in the muOPR spectrum were observed at some wavelengths due to the resulting dilution, but these were negligible at 210 nm (Figure 10). Calculations of binding constants for compounds to the receptor were therefore made using the 210 nm spectral shifts as well as at 200 nm, which required additional corrections for the dilution effect. As expected from the muOPR peptide results, neither histamine (Figure 11) nor acetylcholine (Table 3) nor ascorbic acid (Table 3) bound to intact muOPR with measurable affinity.

Unlike histamine, acetylcholine, and ascorbic acid, met-enkephalin (Met-Enk) produced easily observable shifts in the UV spectrum of the muOPR (Figure 12), as did epinephrine (Figure 13). These results confirm the peptide binding data summarized in Table 2. Very significant differences were apparent in comparing the combination of Met-Enk with epinephrine (Figure 14) and their individual spectra especially in the 190–200 and 220–230 nm ranges. The data from Figure 12 and Figure 13 were used to calculate values for the shifts at both 200 nm and 210 nm that would be expected by adding Met-Enk and epinephrine aliquots together at each step. These figures were then compared with the data obtained by the actual experiment (Figure 14). The binding curves at 210 nm are shown in Figure 15. The observed binding of the combination is shifted to the left by about half a log unit as compared with the binding constants calculated from the individual components. The combination of met-enkephalin with epinephrine results in increased affinity of the muOPR for the compounds as compared with their affinity individually. In other words, the combination enhances binding.

Notably, the 200 nm data reveals what appears to be high affinity binding of Met-Enk to its receptor that is not evident in the 210 nm data (Figure 16). We conclude from this result that binding of the opioid to the receptor involved either different types of interactions that are measurable at 200 nm but not at 210 nm (e.g., displacement of water or ionic bonding) and/or conformational shifts in the receptor that are preferentially observable at 200 nm. In the event, the high affinity binding appears to be enhanced both in terms of its magnitude and a leftward shift in the binding constant in the 200 nm results (Figure 16). It should be noted, however, that the 200 nm results are somewhat less reliable than the 210 nm results because of the additional need to correct for the effects of buffer dilution at 200 nm, which is not a problem at 210 nm (see Figure 10).

**Figure 18:**
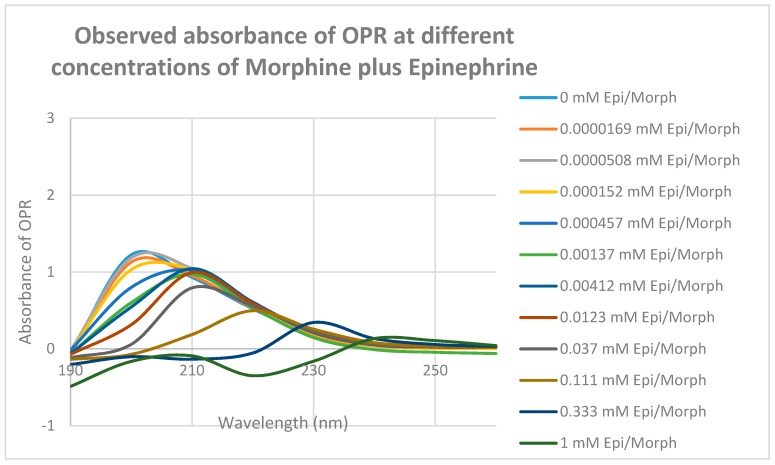
UV spectra of mu opioid receptor (OPR) with serial additions of both morphine and epinephrine (EPI) in tandem. Note the obvious differences from Figure 13 and Figure 17.

**Figure 19:**
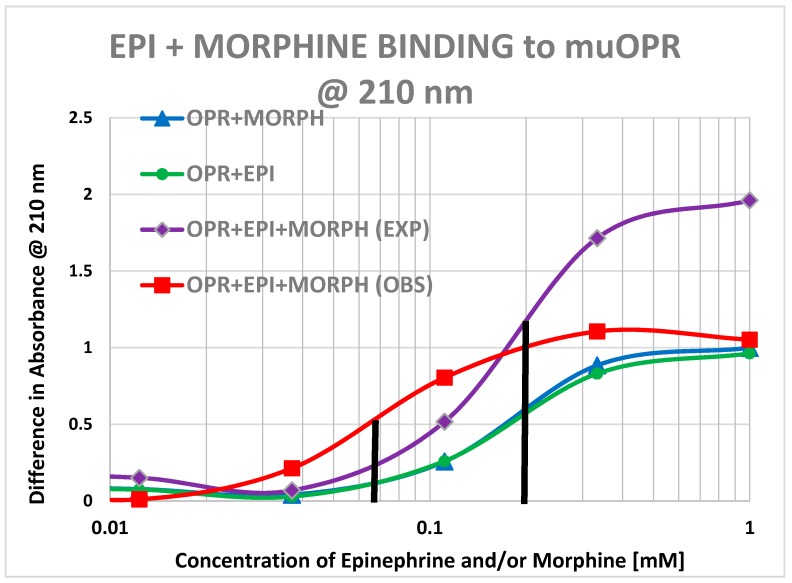
Mu opioid receptor (OPR) binding curves with morphine, epinephrine (EPI) and their combination (MORPH + EPI) at 210 nm. The choice of 210 nm is explained in Figure 10. The experimentally observed binding curve (PR + MORPH + EPI (OBS)) is compared with the theoretically predicted binding calculated from individual binding of MORPH to OPR and EPI to OPR (OPR + MORPH + EPI (EXP)). There is a half-log unit shift to the left (black vertical lines) in the observed binding as compared with the predicted binding.

**Figure 20:**
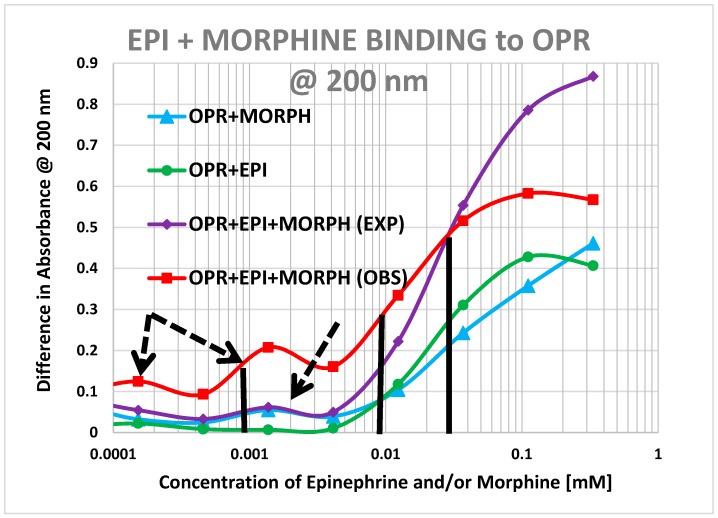
Mu opioid receptor (OPR) binding curves with morphine, epinephrine (EPI) and their combination (MORPH + EPI) at 200 nm. There is a half-log unit shift to the left (black vertical lines) in the observed binding as compared with the predicted binding and the possibility of high affinity binding of morphine to the muOPR appears to be present and is enhanced in the presence of EPI (dashed arrows).

Morphine also produced shifts in the UV spectrum of the muOPR (Figure 17), as with Met-Enk. Visual comparison of Figure 13, Figure 17 and Figure 18 reveal very obvious differences in the spectrum in the 190–200 nm and 220–230 nm ranges. As with the Met-Enk–epinephrine experiment, we used the 200-nm and 210-nm data from morphine binding (Figure 17) and that of epinephrine binding (Figure 13) to calculate values for the shifts that would be expected by adding morphine and epinephrine aliquots together. The expected values at 200 nm and 210 nm were then compared with the data obtained by the actual experiment (Figure 18) and binding curves calculated (Figure 19 and Figure 20). Significant differences were apparent between the expected and experimentally observed values of dual addition of morphine and epinephrine. The observed binding of the combination is, like that observed for met-enkephalin-plus-epinephrine, shifted to the left in both the 200 nm and 210 nm spectra by about half a log unit as compared with the binding constants calculated from the individual components. The combination of epinephrine with morphine enhances binding. As with Met-Enk, there appears to be high-affinity binding of morphine to its receptor, and this high-affinity binding appears to be enhanced in the 200 nm results (Figure 20). Again, as in the Met-Enk case, the high-affinity binding is not evident in the 210 nm results (Figure 19 and Figure 20), suggesting that the type of binding or the regions of the receptor observed by the spectroscopy at 200 and 210 nm differ. In combination with epinephrine, the high-affinity binding of the morphine appears to be enhanced in both magnitude and in a leftward shift of the binding curve, similar to the enhancement observed with Met-Enk. However, once again, the 200 nm results are somewhat less reliable than the 210 nm results because of the additional need to correct for the effects of buffer dilution at 200 nm, which is not a problem at 210 nm (see Figure 10).

Table 3 summarizes the binding constants calculated for these compounds and others tested on the intact, isolated human muOPR.

Notably, since both opioids and epinephrine bind to intact, isolated muOPR, and their binding is synergistic, we conclude that the binding sites for the two compounds are not competitive even though both sets of compounds have some affinity for the extracellular loops. This conclusion is consistent with the main, high-affinity site of opioids being in a pocket formed inside the transmembrane region of the OPR, while epinephrine is limited to a secondary binding site. Thus, the combination experiments on epinephrine binding to intact, isolated muOPR along with the data from the extracellular loop data from Table 2 lead us to conclude that adrenergic agonists bind to extracellular loops 1 and 2 of the muOPR. These experiments are not able to determine whether this pair of extracellular loops cooperate in binding adrenergic agonists or represent two, separate binding sites.

## 3. Discussion

To summarize, our experiments demonstrate that muOPR and ADR manifest similar sequences, especially in their transmembrane regions and extracellular loops. Some of these shared regions of the muOPR, when synthesized as peptides, bind adrenergic compounds. Adrenergic compound binding was also demonstrated to intact, isolated muOPR and such binding synergized with Met-Enk binding and morphine binding to the muOPR. This synergism is evident both in the shift of the binding curves to the left for the low affinity binding site(s) observable at 200 and 210 nm, and also, though with less certainty, in the leftward shift and enhancement of the high affinity binding observable only at 200 nm. Notably, the binding of adrenergic compounds resulted in different shifts in the muOPR spectrum than did binding of opioids, which differed in turn from opioid antagonists. No binding to muOPR or its peptides was observed with acetylcholine, histamine or adrenergic antagonists, and less binding occurred with serotonin to muOPR peptides than with adrenergic compounds.

Our observation that muOPR and ADR share a significant sequence is consistent with work by Wolf and Grünewald [82], who noted that although muOPR are often classified as peptide receptors, they appear to have greater similarities to monoamine receptors. Given that both muOPR and ADR bind both opioid compounds and adrenergic compounds, we suggest that the two classes of receptors may have evolved from a common predecessor and we will be reporting on evidence to support this possibility elsewhere. This possibility is further strengthened by the fact that binding of adrenergic compounds to muOPR is in regions that mimic adrenergic binding regions of ADR (data provided here) and that opioid compounds bind to regions of the ADR that mimic those of the muOPR (Table 2 and [42,43]).

In reviewing the data, we believe that Table 2 demonstrates that there are three types of binding of the compounds we tested to the muOPR: no observable binding (>1000 µM); non-specific binding (30–300 µM); and specific binding (<10 µM). No observable binding can be explained by a lack of appropriate amino acid residues capable of interacting with the compound. Non-specific binding can be explained by the presence of hydrogen and ionic-bond-forming amino acids that do not require the peptide to be in a specific conformation. Specific binding probably requires a particular arrangement of amino acids that form a conformationally-constrained pocket. Any particular muOPR peptide may therefore have high-affinity specific binding for a compound through an induced-fit into a well-defined pocket, but also have a non-specific binding motif made up of the amino acids that do not define that pocket. Because met-enkephalin is also a peptide, there are probably several conformations that permit its interaction with OPR extracellular loop peptides, one of which is high affinity, the other low. This explanation is consistent with the observation that both opioids and adrenergics can bind to the same peptides, yet do not compete in the OPR: opioids are binding only transiently and non-specifically to the extracellular loops before moving into the much higher affinity pocket at the center of the OPR, whereas the loops form the high affinity, specific site for the adrenergics. Thus, in the intact OPR, the high affinity opioid and high affinity adrenergic binding sites do not compete with each other.

The observation that both adrenergic compounds and opioid compounds bind to muOPR extracellular loop peptides is consistent with several types of evidence from other studies. In the first place, the opioid agonist DAMGO utilizes the extracellular loops to distinguish between mu and deltaOPR [83], suggesting that these loops act as initial attractors for OPR ligands. The mutual binding of adrenergics and opioids to extracellular loop peptides and to the intact muOPR is also consistent with Monroe et al.’s [84] study of binding by serotoninergic and adrenergic compounds to muOPR and deltaOPR with low micromolar affinity. Monroe et al. [84] demonstrated that this binding competed weakly with opioid binding, but did not achieve 50% inhibition even at adrenergic or serotoninergic concentrations of 100 μM. Their data, in combination with the fact that both adrenergic and opioid compounds bind to the same muOPR peptides (Table 2) suggests that the extracellular region of the OPR may play a dual role. One role may be to initially attract opioid compounds to the receptor through low-affinity binding. The high-affinity binding site for opioids is known to be located deeper within the OPR, where the ligand is nested in a cavity formed by the transmembrane regions of the receptor [85], which have little or no sequence similarity (Figure 1) to equivalent adrenergic receptor regions. The second role for the extracellular loops of the opioid receptor may be to bind adrenergic, and possibly serotoninergic, agonists. Binding of opioids may facilitate binding of aminergic compounds through allosteric shifts in receptor conformation, or conversely, binding of aminergic compounds may allosterically alter the affinity of the receptor for opioids, or perhaps both effects occur. This low-affinity, combined opioid-adrenergic binding site would explain the partial antagonism of adrenergic and opioid compounds for OPR, also explaining why high-affinity opioid binding is not affected. Wilkerson et al. [86] and Jacobsen et al. [87] report, however, that the high-affinity noradrenergic neurotoxins xylamine and DSP4 both bind to opioid receptors, significantly inhibiting function, so that higher affinity adrenergic antagonists may effectively compete with opioids for opioid receptor binding.

Our observation of increased high-affinity binding of opioids to muOPR (Figure 16 and Figure 20, Table 3) in the presence of epinephrine reported here has also been reported for the adrenergic agonists phenylephrine and isoproterenol [88]. Notably, the enhancement of morphine binding to the muOPR in the presence of clonidine has also been documented but not reported by Jordan et al., [13]. In their Figure 3A (see [13]), they show that there is more than a log unit shift to the left (i.e., increased affinity) of morphine binding to the muOPR when it is in the presence of α-2A ADR, strongly arguing for a positive allosteric effect of ADR on OPR affinity for its ligands. A similar allosteric effect would explain their observation of a similar left-ward shift in pMAPK/ubiquitination of muOPR in the presence of α-2A ADR (Figure 3C in [13]). Most notably, when cells expressing both muOPR and α-2A ADR were exposed to a combination of both morphine and the adrenergic agonist clonidine, there was again at least a log unit leftward shift in binding of both GTPγS and pMAPK as compared with morphine alone (Figure 3B,D in [13]). Unfortunately, these effects were apparently overlooked by Jordan et al. [13] in light of other effects on total binding revealed by the same experiments (which will be discussed below).

**Figure 21:**
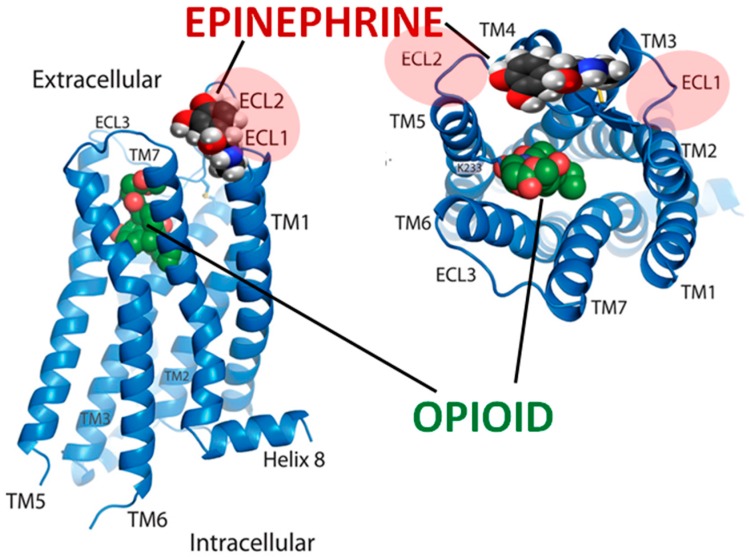
Model for epinephrine binding to the mu opioid receptor, adapted and modified from [85]. The data illustrated in Figure 4, Figure 5, Figure 6, Figure 7 and Figure 8 suggest that adrenergic agonists, but not antagonists or most other compounds (Table 2), bind to portions of both the first extracellular loop (ECL1) and the second extracellular loop (ECL2) but not transmembrane (TM) regions. Opioids also bind to the same extracellular loops suggesting that these loops act as semi-specific attractors for both ligands and enhancers of receptor activity.

Our data, along with the varied literature reports just summarized, support the hypothesis laid out in the Introduction, which was that adrenergic compounds may enhance muOPR activity just as opioids enhance ADR activity. Specifically, it appears that the extracellular loops of the muOPR mimic similar regions in the ADR and specifically recognize both opioid compounds and adrenergic compounds as ligands (Table 2 and Figure 2, Figure 3, Figure 4, Figure 5, Figure 6, Figure 7 and Figure 8). The specific interactions mediating binding of adrenergic compounds to muOPR clearly differ from those mediating binding of opioids and opioid antagonists as demonstrated by the very different shifts in the UV spectra of intact muOPR binding to the various compounds tested in our studies (Figure 10, Figure 11, Figure 12, Figure 13, Figure 14, Figure 17 and Figure 18). These differences in the spectra suggest that different compounds not only bind to different regions of the receptor, but cause different conformational shifts that help to explain their observed differences in action. The binding of opioid compounds to these regions is probably transient as the opioids move into the center of the muOPR where the affinity and specificity are significantly higher. This leaves these extracellular loops free for adrenergic binding, which produces allosteric changes in the muOPR structure that are evident in the spectrophotometric studies conducted here. Such allosteric changes may directly produce the enhanced opioid binding we found (Figure 15, Figure 16, Figure 19 and Figure 20) and/or the adrenergic compounds may “cap” the OPR, trapping the opioid in its binding site, and thereby increasing apparent binding (Figure 21).

The enhancement of opioid binding in the presence of adrenergic agonists is consistent with the many clinical and experimental studies reviewed in the Introduction that have established opioid-adrenergic synergy. This enhancement may be mediated by the OPR and ADR receptors separately or by heterodimers that form between the receptors upon stimulus with their agonists [15,16,17,20,89,90].

An additional phenomenon also sheds important light on the mechanisms involved in synergy. Experiments and clinical studies both demonstrate that combining opioids with adrenergics prevents the development of fade (the rapidly diminished response to repeated doses of a drug) and tachyphylaxis (the longer-term down-regulation of receptors in response to repeated or chronic exposure to a drug) in both ADR and OPR. The enkephalin agonist DAMGO ([d-Ala^2^, *N*-MePhe^4^, Gly-*ol*]-enkephalin) has been shown to reverse both fade and tachyphylaxis due to repeated exposure to catecholamines [39]. Similarly, ascorbic acid (vitamin C) has been shown to reverse fade and prevent tachyphylaxis in guinea pig tracheal smooth muscle and rabbit aorta exposed to adrenergic agonists [70,73]; to prevent tachyphylaxis in rabbit vasculature in vivo [91]; and to reactivate vascular responses to dobutamine and other adrenergic drugs in human subjects [92,93,94,95]. The mechanism of fade and tachyphylaxis reversal almost certainly involves inhibition or reversal of G-protein-mediated phosphorylation by means of allosterically-modulated phosphodiesterase inhibition [43,73,96].

Similarly, epinephrine, clonidine and other adrenergic agonists inhibit the development of tachyphylaxis caused by opiate analgesia [97,98] and epinephrine and dopamine, but not the adrenergic antagonists propranolol or phentolamine, can reverse “acute tolerance” (i.e., fade or tachyphylaxis) caused by repeated doses of morphine on guinea pig ileum [99,100,101]. Since tachyphylaxis reversal is mediated by intracellular G-protein subunits [102], the mechanism of this reversal, like that of catecholamine-induced reversal, would be expected to involve allosteric modification of receptor structure and interference with phosphorylation by receptor kinases. Studies of cells co-expressing OPR and ADR dimerized complexes provide direct evidence of increased receptor activity accompanied by decreased kinase activity and phosphorylation of the receptors in the presence of combinations of morphine and norepinephrine (NE). Jordan et al. [13] reported significantly decreased G-protein γS binding to receptors and significantly decreased pMAPK activity (as surrogate for phosphorylation) in cells co-expressing muOPR along with α2-ADR when exposed to morphine and the adrenergic agonist clonidine as compared with cells exposed to only morphine (Figure 3B, in [13]). Similarly, Vilardaga et al. [16], using FRET experiments, demonstrated immediate allosteric effects of adding clonidine to morphine-activated muOPR that correlated with significantly decreased receptor phosphorylation (Figures 3 and 4 in [16]). Both sets of data strongly support the proposition that the steps involved in receptor down-regulation and internalization that are begun by phosphorylation of the receptors (and therefore initiate fade and tachyphylaxis) are very significantly inhibited in the presence of combinations of opioids and adrenergic agonists.

Oddly, then, both Jordan et al. [13] and Vilardaga et al. [16,17] have concluded that OPR–ADR dimers in the presence of both adrenergic and opioid agonists are allosterically down-regulated. If such down-regulation actually occurred, however, then one would expect combinations of opioids with adrenergic agonists to cause inhibition of activity rather than the synergy that clinical and experimental studies universally report. Also, fade and tachyphylaxis of ADR should not be reversible by opioids, nor fade and tachyphylaxis of OPR by adrenergic agonists; quite the contrary, addition of the second compound should enhance down-regulation. We therefore believe that Jordan et al. [13], and Vilardaga et al. [16] have misinterpreted their own data.

To understand this misinterpretation, we propose a novel model of OPR–ADR synergy that is consistent with the observed synergy of their ligands on individual receptors, the ability of both opioids and adrenergic compounds to reverse fade and tachyphylaxis caused by the other, as well as Jordan et al.’s [13] and Vilardaga et al.’s [16] phosphorylation data. This model is an elaboration of one previously proposed to explain ADR enhancement by opioids and related compounds and their ability to reverse fade and to prevent tachyphylaxis [43,73].

**Figure 22:**
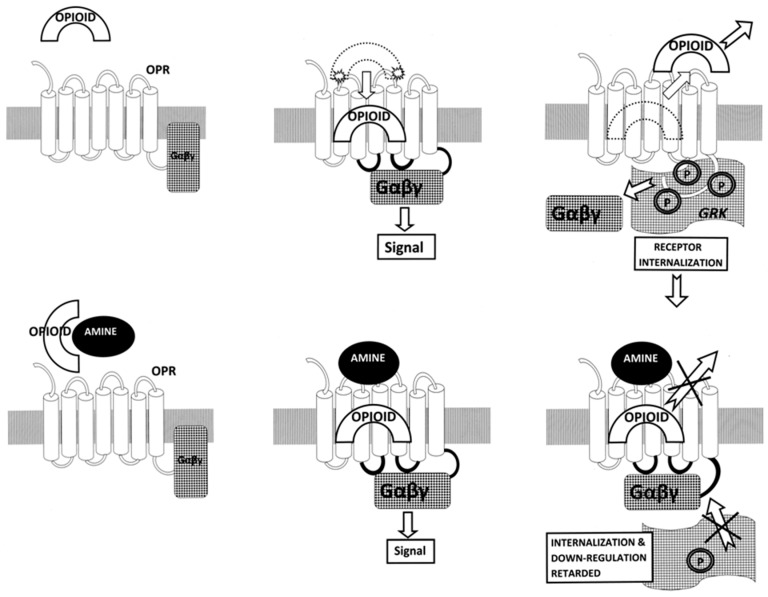
Schematic representation of opioid receptor (OPR) function in the absence and presence of an adrenergic enhancer. Top row from left to right: In the absence of an adrenergic enhancer, an opioid ligand is attracted to the opioid receptor; our data suggest that initial binding to the receptor is to a low-affinity, semi-specific site on the first and second extracellular loops [83], after which the opioid is drawn into the high-affinity, high-specificity cavity formed within the transmembrane loops [85]; high-affinity binding initiates G-protein coupling (Gαβγ) to the intracellular loops of the receptor, followed by the release of the ligand, phosphorylation (P) of the receptor by receptor kinases (GRK), receptor inactivation and internalization. Bottom row from left to right: In the presence of an adrenergic (or possibly serotoninergic) enhancer (“amine”), the same series of steps occur as in the top row, except that the enhancer binds to the extracellular loops after opioid high affinity binding, either “capping and trapping” the ligand in the receptor and/or maintaining the receptor in its high-affinity state for the ligand. In either case, the opioid ligand is not released as quickly from the receptor, preventing the allosteric alterations required for kinase phosphorylation and inactivation of the receptor. The overall effect of enhancer binding is therefore to keep receptor signaling “on” for a longer period of time than occurs in its absence.

Begin with the simple case of cells expressing just OPR. One novel point that our peptide-binding results suggest is that opioid binding to the OPR may be initiated by a low-affinity interaction with the first two extracellular loops of the receptor (Figure 22). This weak interaction then guides the opioid into the high-affinity site within the receptor. The initial binding of opioids to the extracellular loops of the receptor occurs in competition with adrenergic compounds, but the high-affinity binding of the opioids is not affected by the presence of adrenergic compounds. Binding of an opioid in the absence of an adrenergic compounds activates G-protein binding to the intracellular portion of the receptor causing conformational shifts that release the opioid from the receptor. This step is the followed by release of the G-protein cluster and phosphorylation of the receptor (fade) leading to its down-regulation and cellular internalization (tachyphylaxis). At the same time, the β-arrestin2 pathway is activated, which is responsible for many of the respiratory and constipation symptoms that are associated with high or chronic dosages of opioids.

In the presence of an adrenergic agonist, this process is allosterically modified. Binding of the adrenergic agonist in the presence of an opioid results in the opioid being retained in the receptor for a longer period of time, either due to allosteric modifications of receptor structure by the adrenergic compound to keep the OPR in its high affinity state; by “capping” of the opioid binding site, “trapping” the opioid inside the receptor, or both. The effect of opioid retention in the receptor is two-fold. One is to increase the time any given opioid molecule is active in the receptor so that the aggregate activity of any given dose of opioid is enhanced in the presence of the adrenergic compound. Secondly, the allosteric effects of the combined opioid-adrenergic binding prevent release of the G-protein complex and subsequent phosphorylation of the receptor so that activity is maintained at a higher level for a longer period of time than when the adrenergic compounds is absent (Figure 22). The development of fade and tachyphylaxis is therefore retarded.

**Figure 23:**
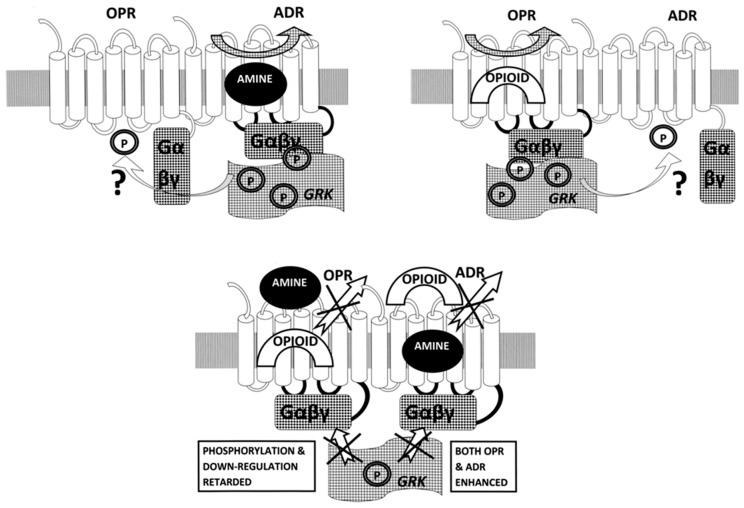
Schematic representation of opioid receptor (OPR) function when dimerized with adrenergic receptor (ADR). Left: In the presence of an adrenergic agonist, only the ADR is activated and the processes of G-protein recruitment (Gαβγ), kinase-mediated (GRK) phosphorylation (P), inactivation of the receptor and its internalization followed as described in [43] and for the OPR in Figure 22. One possible modification of the scheme described for individual receptors is that dimerization may result, through allosteric cross-talk, in inactivation by phosphorylation of the non-activated member of the pair as well. Right: The same process just described for ADR activation, phosphorylation and inactivation will characterize OPR activation in the heterodimer state. Below: Co-activation of both the ADR and OPR in their heterodimerized state will have very different effects than activation of each receptor independently. Both receptors will be enhanced (the ADR by opioids and the OPR by adrenergics), preventing release of the ligand, maintaining signaling and inhibiting (indicated by Xs in the figure) phosphorylation and internalization of both receptors. Allosteric cross-talk may further enhance the continued activation of the receptor pair in the dimerized state. This model explains clinical and experimental observations of adrenergic-opioid synergy and prolongation of activity, as well as inhibition of receptor phosphorylation when both compounds are present.

Since most cells and tissues expressing OPR also express ADR, and these receptors may form heterodimers, the actual situation is more complex than just described. As described previously by Root-Bernstein and Dillon [43,73], the ADR is affected by the presence of opioid compounds in the same way that the OPR is affected by the presence of adrenergic agonists. Thus, both the OPR and the ADR are enhanced simultaneously (Figure 23). Moreover, activation of either the OPR or the ADR can produce OPR–ADR dimers that can allosterically modify each other’s activity. The presence of both opioid and adrenergic ligands will therefore enhance dimer formation as well as the activation of both receptors. The consequence will be to inhibit phosphorylation of the receptors, maintaining both OPR and ADR activity for much longer periods of time at much lower concentrations of each compound. This model also provides a mechanism for reversal of fade or tachyphylaxis since the same allosteric alterations in the receptors (and their dimers) will reverse G-protein-mediated processes and kinase-mediated phosphorylation (Figure 23).

The reasons that Jordan et al. [16] and Vilardaga et al. [13] observed decreased GTPγS and pMAPK activity in the presence of opioid-adrenergic combinations as compared with either compound alone follow directly from the model. The model, however, interprets these effects not as decreases in receptor activity (as Jordan [13] and Vilardaga [16] did) but rather as evidence that the receptors maintain their activity, resisting the processes that would normally release the ligands and initiate receptor internalization. This re-interpretation of the Jordan and Vilardaga data is consistent with the clinical and experimental observations of opioid-adrenergic synergy summarized in the Introduction, whereas the decreased-activity models proposed by Jordan and Vilardaga are not [13,16,17].

If the model proposed above is correct, and both fade and/or tachyphylaxis due to exposure to opioids can be reversed by adrenergic agonists, then new drug development options exist for producing what are currently being called “biased opioids” that provide powerful pain relief without activating the β-arrestin2 pathway. Such “biased opioids” produce less respiratory depression and constipation [103] and are therefore considered safer than standard opioids. One possibility that follows from our results is that of synthesizing a tethered compound comprised of an adrenergic subunit and an opiate subunit that would specifically enhance a particular class of opioid receptors. Such a compound might be expected to require lower dosages than current opioids, increased longevity or action and lower side effects that existing opioid drugs. A similar approach previously led from the discovery of the fact that ascorbic acid enhances and prolongs adrenergic activity to the development a linked compound comprised of an adrenergic subunit and ascorbic acid, which specifically targeted adrenergic receptors [104]. Such linked compounds can be expected to incorporate the enhanced binding and activity or the components without the general systemic side effects of the components. An alternative possibility would to link an opiate agonist to an adrenergic antagonist to limit OPR enhancement. Linking opiate antagonists with adrenergic agonists or antagonists would open other therapeutic options. Furthermore, development of such linked compounds would also provide an additional means of investigating the adrenergic binding site that we describe here on the muOPR and differentiating it from the opiate-enhanced adrenergic site on ADR. The types of linked compounds we are suggesting are not to be confused with those bivalent ones under consideration for stabilizing homo- and heterodimerized receptors (e.g., [105]), which can be expected to have quite different effects due to the mutual allosteric regulation of ADR and OPR for each other in the dimerized state.

We also note, in conclusion, that serotoninergic compounds have been reported to have similar opioid enhancing effects in several of the papers cited here (e.g., [67,69,84]), so that serotonin should be investigated for OPR binding and synergy more fully and its therapeutic possibilities more fully explored.

## 4. Materials and Methods

### 4.1. muOPR-ADR Similarity Search

Our initial approach to our hypothesis was to examine whether adrenergic receptors and opioid receptors share significant regions of similarity. A variety of human α and β adrenergic receptor and human opioid receptor sequences were identified in the UniProt database (Available online: www.expasy.org). These were compared using LALIGN (Available online: www.expasy.org), BLOSSUM80, local search, all parameters set to default values [106].

### 4.2. Opioid Receptor Peptide Synthesis and Preparation

Five peptides from the muOPR with varying degrees of similarity to the ADR were synthesized to at least 95% purity (as determined by mass spectrometry) by RS Synthesis (Louisville, KY, USA): Mu 38–51, Mu 111–122, Mu 121–131, Mu 132–143, and Mu 211–226 (sequences provided in Table 1). Each of these receptor peptides were made into individual stock solutions with a concentration of 1 mg/mL in pH 7.40 phosphate buffered saline solution (Fischer Scientific, Hampton, NH, USA). For each individual test, the stock solution was diluted to 0.1 or 0.2 mg/mL solution. Each of these peptide solutions was tested for binding with various adrenergic compounds and controls such as histamine and acetylcholine (Sigma-Aldrich, St. Louis, MO, USA). The adrenergic or compounds were made up at 1.0 mM solutions and then serially diluted by thirds eleven times using phosphate buffer. Results were compared with binding to extracellular loop peptides from the β-2-adrenergic receptor, dopamine receptor, histamine receptor, and insulin receptor (sequences provided in Table 1).

### 4.3. Opioid Peptide Binding Test Methods

After the solutions were made, a 96-well quartz crystal plate was prepared to be run through the spectrophotometer at room temperature (approximately 24 °C). The plate was set up to have the absorbance of each of the adrenergic compound dilutions measured on their own, and with each receptor peptide. The absorbance of each receptor peptide without the presence of the adrenergic compound was measured as well. The absorbance of each well was measured at every 10-nm increment from 190–260 nm. The maximum absorbance that can be measured was set to be 4. Each well had 200 μL of solution, so if the absorbance of one component was being measured, it was diluted by half with phosphate buffer.

Spectrophotometry (SPECTRAmax plus scanning spectrophotometer with the SOFTmax PRO program (Molecular Devices, San Jose, CA, USA) was used to measure the binding between opioid receptor peptides and opioid, adrenergic or control compounds. Beer’s Law shows that if two compounds do not interact in solution, then the absorbance of that solution is equal to the additive absorbance of each of the compounds in solution on their own. The binding between two compounds at a specific wavelength is found using the difference of the additive absorbances of each of the compounds in solution on their own, and the absorbance found when they are in solution together. If the measured absorbance is different than the additive absorbance of each compound, then that indicates some sort of molecular interaction. The binding can be quantified by graphing the difference in absorbance against the concentration of the compound varied to provide a binding curve. The absorbance for the phosphate buffer is subtracted for each well before any calculations are done. The data are analyzed by finding the additive absorbance, and plotting the difference between this absorbance and the actual absorbance against the concentration of the adrenergic compound. All data were analyzed using Microsoft Excel (Microsoft, Inc., Redmond, WA, USA), which reveals an “S” binding curve if binding is present. The binding constants were estimated from the half-saturation point at 200 nm since the absorbance differences tended to be maximized at this wavelength. Calculation of the binding constants at 195 through 215 yielded identical results but the quality of the curves were sometimes degraded.

### 4.4. Human Mu-Opioid Receptor (muOPR) Expression and Purification

Codon optimized human mu opioid receptor gene with N-terminal deca-histidine tag in pQE-2 vector was used for protein expression in *E. coli* [80]. MuOPR expression was achieved using the muOPR transformed C43 (DE3) cell strain of *E. coli* in Terrific Broth medium as reported earlier [80] with 0.4 mM isopropyl β-d-1-thiogalactopyranoside induction at 18 °C for 24 h. Bacterial cell cultures were harvested by centrifugation at 4000× *g* for 20 min. Periplasmic fraction from the harvested cells was removed by osmatic shock [81]. Cells were resuspended in 7 mL of lysis buffer (20 mM Tris-HCl pH 8.0, 150 mM NaCl, 10% glycerol, 2 mM MgCl_2_, 10 μM E-64, 1 μM pepstatin-A, 10 μM leupeptin, 1 mM pefabloc SC, 2 mM β-Mercaptoethanol, 1 mg/mL lysozyme, 30 U/mL DNase) per gram of cell pellet and incubated on ice for 30 min with continuous stirring. The partial lysate was added with EDTA to a final concentration of 5 mM and passed through a high pressure homogenizer EmulsiFlex-C3 (Avestin Europe GmbH, Weinheimer Str. 64b, 68309 Mannheim, Germany) 2–3 times for efficient cell lysis. The lysate was clarified by centrifugation at 10,000× *g* for 40 min at 4 °C. Supernatant was collected to isolate the membrane fraction by centrifuging at 100,000× *g* for 1 h at 4 °C. The isolated membrane was solubilized in 20 mL of solubilization buffer (20 mM Tris–HCl pH 8, 300 mM NaCl, 10% Glycerol, 1% Fos-12, 10 μM E-64, 1 μM pepstatin-A, 10 μM leupeptin, 1 mM pefabloc SC (Sigma-Aldrich, St. Louis, MO, USA)) per gram of membrane for 3 h at 5 °C (cold room) with continuous stirring. The solubilized membrane sample was centrifuged at 100,000× *g* for 1 h at 4 °C and the supernatant was collected. Imidazole was added to the supernatant to a final concentration of 5 mM before starting purification using Ni-NTA resin in batch mode as reported [80]. Eluted fractions with muOPR were pooled, concentrated and subjected to size exclusion chromatography (SEC) using HiLoad 16/600 Superdex 200 pg column (GE Healthcare Sciences, Berlin, Germany) equilibrated with 20 mM Tris pH 8, 150 mM NaCl, 0.1% Fos12, 10% Glycerol and 1 mM TCEP. SEC elution fractions were analyzed by SDS-PAGE and western blotting with Monoclonal Anti-polyHistidine-Peroxidase antibody (Sigma-Aldrich) for protein presence and purity before use. The estimated amount of muOPR in the pure sample was 309 μg in 3 mL.

### 4.5. Binding of Epinephrine and Opioids to muOPR Monitored by Ultraviolet Spectroscopy

A stock solution of 1200 μL of muOPR was formulated using 600 μL of muOPR (0.103 mg/mL) and 600 μL of 20 mM Tris buffer (pH 8). 500 μL of 20 mM Met-Enkephalin (Sigma-Aldrich), morphine sulphate (Sigma-Aldrich), epinephrine HCl (Sigma-Aldrich), or other compounds tested, were freshly made with 20 mM Tris buffer and subjected to twelve serial dilutions by thirds in the buffer. 100 μL of muOPR were pipetted into twelve wells of a crystal 96-well plate and 100 μL of buffer were pipetted into an additional twelve wells of the plate. Three muOPR and three buffer wells then received 10 μL of buffer; three received 5 μL of buffer plus 5 μL of epinephrine; three received 5 μL of buffer plus 5 μL opioid; three received 5 μL opioid plus 5 μL epinephrine. The spectrum of the wells was then recorded from 190 to 260 nm using a Spectramax plus scanning spectrophotometer (Molecular Devices, San Jose, CA, USA) using SOFTMax PRO software (Molecular Devices, San Jose, CA, USA). The procedure described above was repeated an additional eleven times using compound dilutions of increasing concentration each time.

Data were analyzed in Excel. The raw spectra were processed by averaging the three runs of each condition and then subtracting the absorbance of the buffer alone at each volume. The triplicate data for each experimental condition were averaged. The compound + buffer data were subtracted from the muOPR + compound data at each volume to leave the spectrum of the muOPR under that experimental condition. The difference between the muOPR under that experimental condition and the muOPR merely diluted with the same volume of buffer was then calculated and this data used to calculate the binding constant of the compound for muOPR. Because the final calculations involve several subtractions, error bars could be calculated.

## Figures and Tables

**Table 1:** List of peptides synthesized from extracellular and transmembrane loops of the mu opioid receptor (muOPR), β-2-adrenergic receptor (B2AR), dopamine receptor D1 (D1DR), histamine 1 receptor (Hist Rec) and insulin receptor (Insulin Rec).

MuOPR 38–51	DGNLSDPCGPNRTD
MuOPR 111–122	NLALADALATST
MuOPR 121–131	TLPFQSVNYL
MuOPR 132–143	MGTWPFGTILCK
MuOPR 211–226	KYRQGSIDCTLTFSHP
B2AR 93–100	HILMKMWT
B2AR 97–106	KMWTFGN
B2AR 103–113	NFWCEFTSID
B2AR 175–188	RATHQEAINCYANE
D1DR 89–100	FWPFGSFCNWV
D1DR 175–188	ATSLAETINCDS
Hist Rec 77–87	GAVVMPMNILYL
Hist Rec 89–98	LMSKWSLGRP
Hist Rec 96–107	RPLCLFWLSMD
Hist Rec 105–115	SMDYVASTASI
Hist Rec 166–172	NHFMQQT
Hist Rec 177–183	RDKCETD
Insulin Rec 105–113	NLTVIRGSR
Insulin Rec 157–166	TIDWSRILDS

**Table 2:** Binding constants (in micromoles) of various bioactive compounds for the receptor peptides listed in Table 1.

**Kd (µM) @ 200 nm**	**Morph**	**Nalox**	**MENK**	**Epi**	**NorEpi**	**Amph**	**DOP**	**L-DOPA**	**Salb**	**Prop**
Mu 38–51	35	0.5/35	0.15/55	1.2/35	1.4/45	1.25/90	60	50	33	250
Mu 111–122	50	0.5/38	0.33/80	1.25/40	1.3/40	1.3/100	65	60	25	300
Mu 121–131	900	>1000	3.5/90	>1000	>1000	>1000	>1000	>1000	>1000	>1000
Mu 132–143	35	0.5/42	0.4 /70	1.4/35	1.35/40	1.1/85	60	50	35	150
Mu 211–226	30	1.0/45	1.0/65	1.2/40	1.3/45	1.2/90	65	60	33	250
B2AR 93–100	8	5								
B2AR 97–106	1	6								
B2AR 103–113	27	3								
B2AR 175–188	50	40								
D1DR 89–100	20	5								
D1DR 175–188	10	20								
HIST 77–87	70	30								
HIST 89–98	5	4								
HIST 96–107	15	10								
HIST 105–115	50	40								
HIST 166–172	0.6/30	130								
HIST 177–183	30	150								
INSR 105–113	10	20								
INSR 157–166	60	200								
**Kd (µM) @ 200 nm**	**Yoh**	**Phen**	**5HT**	**Mel**	**ACh**	**Hist**	**EDTA**	**ASC**	**RIBO**	**GLUC**
Mu 38–51	300	>1000	100	100	>1000	>1000	900	>1000	50	>1000
Mu 111–122	400	>1000	100	150	>1000	>1000	950	>1000	>1000	>1000
Mu 121–131	>1000	>1000	350	900	>1000	>1000	40	>1000	>1000	>1000
Mu 132–143	250	>1000	100	100	>1000	>1000	700	>1000	100	>1000
Mu 211–226	3.3/250	>1000	90	100	>1000	>1000	900	>1000	>1000	>1000
B2AR 93–100							300	60	400	>1000
B2AR 97–106							50	60	600	>1000
B2AR 103–113							>1000	150		>1000
B2AR 175–188							350	300	500	>1000
D1DR 89–100							900	300	600	>1000
D1DR 175–188							50	300	300	>1000
HIST 77–87							60	300	>1000	>1000
HIST 89–98							20	30	400	>1000
HIST 96–107							90	40	500	>1000
HIST 105–115							60	300	450	>1000
HIST 166–172							250	350		>1000
HIST 177–183							20	40		>1000
INSR 105–113							>1000	>1000	500	>1000
INSR 157–166							>1000	>1000		>1000

Receptors: Mu opioid receptor (Mu); β-2-adrenergic receptor (B2AR); dopamine receptor D1 (D1DR); histamine 1 receptor (HIST); Insulin receptor (INSR). Bioactive compounds: morphine sulfate (Morph); naloxone (Nalox); methionine enkephalin (MENK); epinephrine HCl (Epi); norepinephrine HCl (NorEpi); amphetamine (Amph); dopamine (DOP); l-3,4-dihydroxyphenylalanine (l-DOPA); salbutamol (Salb); propranolol (Prop); yohimbine (Yoh); phentolamine (Phen); serotonin or 5-hydroxytryptamine (5HT); melatonin (Mel); acetylcholine (ACh); ethylenediaminetetraacetic acid (EDTA); ascorbic acid or vitamin C (ASC); riboflavin (RIBO); glucose (GLUC).

**Table 3:** Binding constants (in micromoles) of bioactive compounds, alone and in combinations, to the intact, purified mu opioid receptor (muOPR) measured at 200 and 210 nm.

Compound	Kd @ 200 nm (μM)	Kd @ 210 nm (μM)
Acetylcholine	>1000	>1000
Histamine	>1000	>1000
Ascorbic Acid (Vitamin C)	>1000	>1000
Epinephrine	30	20
Met-Enkephalin	15/0.8	12
Met-Enkephalin + Epinephrine	4/<0.01?	5
Morphine	60/0.9	20
Morphine + Epinephrine	9/0.9/<0.01?	6

Note that at 200 nm, high affinity binding becomes apparent between opioids and the muOPR that are not apparent at 210 nm. In the Met-Enk, Met-Enk + Epi, Morhpine, and Morphine + Epi cases, multiple binding constants are calculated for high affinity and low affinity binding sites (see Figure 16 and Figure 20). Question marks indicate that the highest affinity binding constants are approximated (see far left arrows in Figure 16 and Figure 20).
